# Denosumab improves clinical manifestations of hypophosphatemic osteomalacia by adefovir-induced Fanconi syndrome: a case report

**DOI:** 10.1186/s13256-019-2018-7

**Published:** 2019-04-20

**Authors:** Tomohisa Kunii, Toshie Iijima, Teruo Jojima, Masanori Shimizu, Masato Kase, Shintaro Sakurai, Takahiko Kogai, Isao Usui, Yoshimasa Aso

**Affiliations:** 1 Department of Endocrinology and Metabolism, Dokkyo Medical University, Mibu, Shimotsuga, Tochigi, 321-0293 Japan; 2 Department of Infection Control and Clinical Laboratory Medicine, Dokkyo Medical University, Mibu, Tochigi Japan

**Keywords:** Adefovir dipivoxil, Fanconi syndrome, Osteomalacia, Osteoporosis, Chronic hepatitis B, Denosumab

## Abstract

**Background:**

Adefovir dipivoxil is a nucleotide analogue that is approved for treatment of chronic hepatitis B. Adefovir dipivoxil is associated with proximal tubular dysfunction, resulting in Fanconi syndrome, which can cause secondary hypophosphatemic osteomalacia. We describe a case of a patient with hypophosphatemic osteomalacia secondary to Fanconi syndrome induced by adefovir dipivoxil concomitantly with osteoporosis in whom clinical symptoms were improved by adding denosumab (a human monoclonal antibody targeting the receptor activator of nuclear factor-κB ligand) to preceding administration of vitamin D_3_.

**Case presentation:**

A 60-year-old Japanese man had been receiving low-dose adefovir dipivoxil (10 mg/day) to treat chronic hepatitis B for approximately 5 years. He presented to an orthopedic surgeon with severe pain of the right hip and no trauma history, and fracture of the neck of the right femur was identified. In addition, ^99m^Tc-hydroxymethylene diphosphate scintigraphy revealed significantly abnormal uptake in the bilateral ribs, hips, and knees, and he was therefore referred to our university hospital for evaluation of multiple pathological fractures. We diagnosed hypophosphatemic osteomalacia due to Fanconi syndrome induced by adefovir dipivoxil therapy. Although we reduced the patient’s adefovir dipivoxil dose and added calcitriol (active vitamin D_3_), he did not respond and continued to complain of bone pain. Several bone resorption markers and bone-specific alkaline phosphatase were also persistently elevated. Therefore, we added denosumab to vitamin D_3_ supplementation for treatment of excessive bone resorption. Two months after initiation of denosumab, his hip and knee pain was relieved, along with a decrease in serum alkaline phosphatase and some bone resorption markers.

**Conclusions:**

Although denosumab is not generally an appropriate treatment for acquired Fanconi syndrome, it may be useful for patients who have hypophosphatemic osteomalacia due to adefovir dipivoxil-induced Fanconi syndrome associated with excessive bone resorption. However, clinicians should keep in mind that if denosumab is administered to patients with hypophosphatemic osteomalacia accompanied by excessive bone resorption, adequate vitamin D and/or phosphate supplementation should be done before administration of denosumab.

## Background

Adefovir dipivoxil (ADV) is an acyclic nucleotide analogue of adenosine monophosphate that is widely used to treat chronic hepatitis B. There is considerable evidence that long-term administration of ADV, even at a low dose, causes renal tubular dysfunction due to nephrotoxicity [1–3]. Fanconi syndrome can be induced by generalized proximal tubular dysfunction due to ADV therapy, resulting in hypophosphatemic osteomalacia with pathological fractures [1–3].

Denosumab is a human monoclonal antibody targeting the receptor activator of nuclear factor-κB ligand (RANKL) that is employed for the treatment of osteoporosis [4]. In general, denosumab would not be considered appropriate for patients with hypophosphatemic osteomalacia due to Fanconi syndrome, because denosumab rapidly inhibits osteoclast-dependent bone and calcium resorption and subsequently induces hypocalcemia. However, we encountered a 60-year-old man with hypophosphatemic osteomalacia due to ADV-induced Fanconi syndrome in whom clinical manifestations improved after denosumab was added to vitamin D_3_ supplementation, as reported here.

## Case presentation

**Fig. 1:**
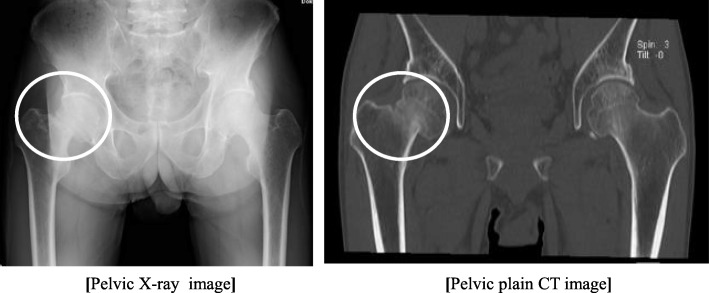
Pelvic x-ray and computed tomography. The *circles* show a fracture of the right femoral neck

**Fig. 2:**
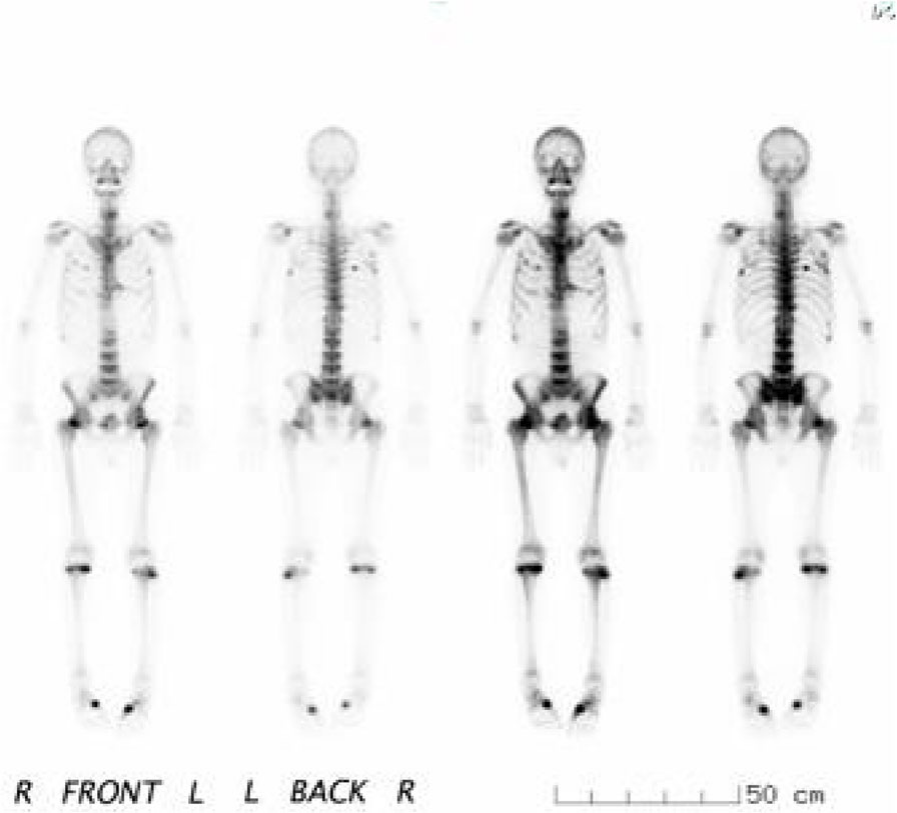
^99m^Tc-hydroxymethylene diphosphate scintigraphy showing increased uptake throughout the skeleton (ribs, hips, and knees)

A 60-year-old Japanese man was referred to our hospital for evaluation of severe bone pain and pathological fracture of the neck of the right femur. He had been receiving treatment for chronic hepatitis B with lamivudine (100 mg/day) and ADV (10 mg/day) since December 2006. In June 2013, he noticed low-back pain and then developed severe pain in the right hip. One month later, he also developed pain of the great toe during walking and was referred to an orthopedic surgeon at our hospital. Fracture of the neck of the right femur was found, despite no history of trauma (Fig. 1). In addition, ^99m^Tc-hydroxymethylene diphosphate scintigraphy revealed significantly abnormal uptake in the bilateral ribs, hips, and knees (Fig. 2). In August 2013, he was referred to our outpatient clinic for evaluation of multiple pathological fractures.

On examination, his body mass index was 18.0 kg/m^2^, temperature was 36.7 °C, blood pressure was 151/86 mmHg, and pulse rate was 67 beats/min (regular). He had generalized bone pain and gait disturbance. His past medical history was appendicitis in 1967 and stomach polyps in 2011. In his family medical history, there was pancreatic cancer, but there was no liver disease. His regular medications were adefovir and ursodeoxycholic acid. He had smoked three packs of cigarettes per day for 30 years, but he had quit since 51 years old. He drinks 350 ml/day of beer. Laboratory tests showed marked elevation of alkaline phosphatase (ALP) (1223 U/L), as well as hypophosphatemia (1.9 mg/dl) and mild hypocalcemia (8.5 mg/dl). His serum creatinine was slightly elevated, whereas serum 1α,25(OH)_2_ vitamin D_3_ was relatively low at 26.4 pg/ml (reference range, 20.0–60.0 pg/ml) (Table 1).

**Table 1:** Laboratory data on admission

			Reference range
Chemistry			
AST	12	U/L	13–30
ALT	7	U/L	10–42
T-Bil	0.5	mg/dl	0.4–1.5
LDH	141	U/L	124–222
ALP	1223	U/L	106–322
ALP1	3	%	
ALP2 + ALP3	91	%	
ALP5	6	%	
LAP	49	U/L	30–70
gGTP	12	U/L	13–64
ChE	267	U/L	215–464
TP	6.6	g/dl	6.6–8.1
Alb	4.1	g/dl	4.1–5.1
BUN	14	mg/dl	8.0–20.0
Cre	1.44	mg/dl	0.65–1.07
eGFR	40.2	ml/min/1.73m^2^	
UA	1.5	mg/dl	3.7–7.0
Na	143	mEq/L	138–145
K	4	mEq/L	3.6–4.8
Cl	113	mEq/L	101–108
Ca	8.5	mg/dl	8.8–10.1
IP	1.9	mg/dl	2.7–4.6
Glucose	93	mg/dl	55–110
HbA1c	4.8	%	4.3–5.8
CRP	0.1	mg/dl	≤ 0.14
Serum β_2_-microglobulin	3.5	mg/dl	
Hepatitis marker			
Hbs-Ag	250	IU/ml	< 0.05
HBs-Ag	0.4	S/CO	< 1.0
HBe-Ab	99.6	%Inh	< 50.0
Hematology			
WBC	5200	/μl	3300–8600
RBC	447	10^4^/μl	4.35–5.55
Hb	16.5	g/dl	13.7–16.8
Hct	47.6	%	40.7–50.1
Plt	16.3	10^4^/μl	15.8–34.8
Bone metabolic parameters			
TRACP-5b	781	mU/dl	170–590
Bone ALP	112	μg/L	3.7–20.9
Deoxypyridinoline	6.7	nmol/mmol Cre	2.1–5.4
U-NTx	216.1	nmol BCE/mmol Cr	13.0–66.2
calcitonin	16	pg/ml	< 9.52
intact-PTH	43.6	pg/ml	8.7–79.6
PTHrP	< 1.1	pmol/L	< 1.1
1,25(OH)_2_ Vit.D3	26.4	pg/ml	20.0–60.0
FGF23	< 5	pg/ml	
Immunology			
Antinuclear antibody	20	times	< 20
C3	74	mg/dl	65–135
C4	13	mg/dl	13–53
CH50	41	U/ml	30–50
IgG	1031	mg/dl	680–1620
IgA	301	mg/dl	84–438
IgM	87	mg/dl	57–288
IgE	3	IU/ml	< 295
Serum immunoelectrophoresis	M protein (−)		
Urinary immunoelectrophoresis	BJ protein (+)		
Blood gas analysis			
pH	7.328		7.350–7.450
PCO_2_	40.3	mmHg	35.0–45.0
PO_2_	110	mmHg	85.0–105.0
HCO_3_-	20.5	mmol/L	23.0–28.0
BE	−4.7	mmol/L	−4.6
AG	4.8	mmol/L	8.0–12.0
Urinalysis			
pH	6.5		
U-glucose	100	mg/dl	
U-blood	–		
BJ-protein	+		
U- total protein	1.3	g/g Cr	
Urinary NAG	7.8	IU/L	0.3–11.5
	16.3	IU/g Cr	
Urinary β_2_-microglobulin	138,885	μg/g Cr	
Na	73	mEq/L	
K	24	mEq/L	
Cl	91	mEq/L	
Ca	29.4	mg/dl	
IP	43	mg/dl	
UA	30	mg/dl	
Cre	48	mg/dl	
%TRP	41.59	%	
FEUA	46.3	%	

*Abbreviations: AG* Anion gap, *Alb* Albumin, *ALP* Alkaline phosphatase, *ALT* alanine aminotransferase, *AST* aspartate aminotransferase, *BCE* Bone collagen equivalents, *BE* Base excess, *BJ protein* Bence-Jones protein, *BUN* Blood urea nitrogen, *CH50* Total hemolytic complement, *ChE* Cholinesterase, *Cre* Creatinine, *CRP* C-reactive protein, *eGFR* Estimated glomerular filtration rate, *FEUA* Fractional excretion of uric acid, *FGF23* Fibroblast growth factor 23, *gGTP* γ-Glutamyl transpeptidase, *Hb* Hemoglobin, *HbA1c* Hemoglobin A1c, *HBe-Ab* Hepatitis B e antigen antibody, *Hbs-Ag* Hepatitis B surface antigen, HCO_3_^−^ Bicarbonate, *Hct* Hematocrit, *Ig* Immunoglobulin, *IP* inorganic phosphorus, *LAP* Leukocyte alkaline phosphatase, *LDH* Lactate dehydrogenase, *NAG* N-acetyl-β-D-glucosaminidase, *NTx* Cross-linked N-telopeptide of type I collagen, *PCO*_*2*_ Partial pressure of carbon dioxide, *Plt* Platelets, *PO*_*2*_ Partial pressure of oxygen, *PTH* Parathyroid hormone, *PTHrP* Parathyroid hormone-related protein, *RBC* Red blood cells, *T-Bil* Total bilirubin, *TP* Total protein, *TRACP-5b* Tartrate-resistant acid phosphatase 5b, *%TRP* Percentage tubular reabsorption of phosphate, *UA* Urinalysis, *WBC* White blood cells

Urinalysis showed glycosuria (2+) and proteinuria (1+). Urinary β_2_-microglobulin was markedly elevated at 138,885 μg/g creatinine (Cr), and tubular reabsorption of phosphate was significantly decreased to 41.59% (reference range for percentage tubular reabsorption of phosphate, 80–94%) (Table 1). On the basis of these results, we diagnosed hypophosphatemic osteomalacia secondary to Fanconi syndrome caused by ADV therapy.

Dual-energy X-ray absorptiometry showed an extremely low bone mineral density with a mean lumbar T-score of − 3.6 SD. Several bone resorption markers were highly elevated (urinary cross-linked N-telopeptide of type I collagen, 216.1 nmol bone collagen equivalents/mmol; urinary deoxypyridinoline, 6.7 nmol/mmol Cr; serum tartrate-resistant acid phosphatase 5b, 781 mU/dl) (Table 1). Taken together, these findings suggested that the patient had excessive bone resorption combined with hypophosphatemic osteomalacia.

**Fig. 3:**
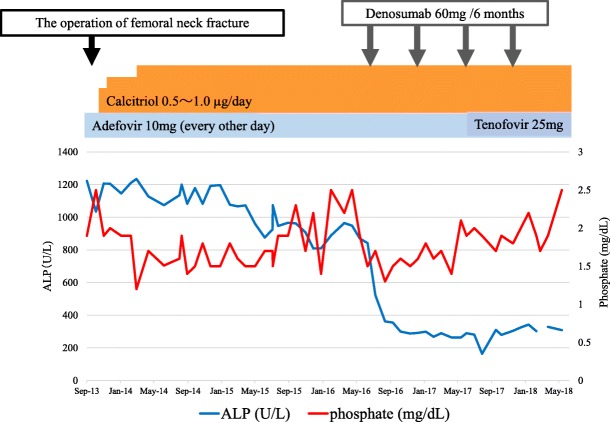
Clinical time course during treatments

**Fig. 4:**
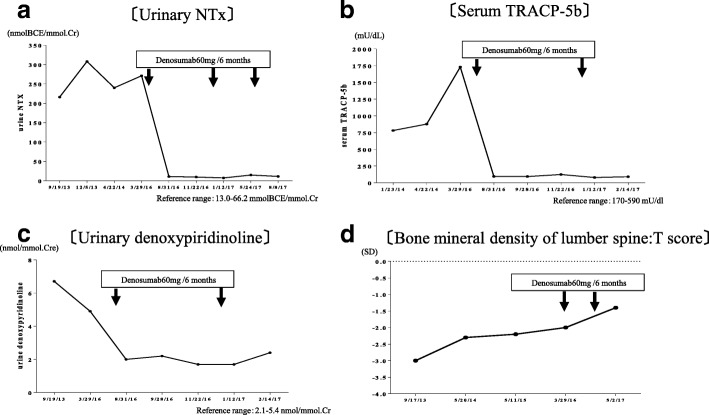
Changes in several bone metabolic markers after treatment with denosumab. **a** Urinary cross-linked N-telopeptide of type I collagen. **b** Serum tartrate-resistant acid phosphatase 5b. **c** Urinary deoxypyridinoline. **d** Bone mineral density of lumbar spine: T-score by dual-energy X-ray absorptiometry. *Note*: Time lines (*x*-axes) are different in each of the graphs

To treat his condition, we first reduced the dose of ADV from 10 mg daily to 10 mg every other day and administered calcitriol (1.0 μg/day) because he had both hypophosphatemia and mild hypocalcemia. In October 2013, he underwent prosthetic replacement of the head of the right femur. However, his generalized bone pain was not relieved by these measures, and several bone resorption markers remained very high, as did serum ALP despite treatment for osteomalacia. In June 2016, we added denosumab (60 mg subcutaneously), a human monoclonal antibody that inhibits RANKL, to ongoing vitamin D therapy in an attempt to suppress persistently high bone resorption. Two months after initiation of denosumab, his hip and knee pain were relieved, along with a decrease in serum ALP and several bone resorption markers (Figs. 3 and 4a–c). Urinary β_2_-microglobulin decreased gradually after addition of denosumab to vitamin D_3_. After 9 months of denosumab treatment, the patient’s mean lumbar T-score increased from − 2.0 SD to − 1.4 SD (Fig. 4d). We administered denosumab 60 mg every 6 months, and currently he continues to receive denosumab.

## Discussion and conclusions

We present a case of a 60-year-old man who had hypophosphatemic osteomalacia secondary to acquired Fanconi syndrome caused by low-dose ADV therapy (10 mg/day). Osteomalacia is a metabolic bone disease characterized by a defective mineralization of the osteoid matrix synthesized by osteoblasts, leading to an accumulation of nonmineralized bone. Osteomalacia is usually associated with vitamin D deficiency or hypophosphatemia. Fanconi syndrome results from generalized dysfunction of the proximal renal tubules, which results in impaired reabsorption of amino acids, glucose, uric acid, bicarbonate, and phosphate, with increased urinary excretion of these solutes [5]. Thus, hypophosphatemic osteomalacia can occur in patients with Fanconi syndrome.

ADV is a nucleotide analogue of adenosine monophosphate that is widely used for the treatment of chronic hepatitis B. The mechanisms by which ADV induces nephrotoxicity remain to be determined, but ADV may impair tubular transport, increase apoptosis, or cause mitochondrial injury in the renal tubular epithelium [6]. It has been reported that ADV-related nephrotoxicity is dose-dependent, with a low dose of ADV (10 mg/day) generally being safe and well tolerated [7]. However, there is evidence that even low-dose ADV can induce nephrotoxicity, including Fanconi syndrome, especially in Asian patients [2, 3, 8, 9]. Recently, there has been an increase of reports regarding Fanconi syndrome associated with long-term ADV therapy, even at low doses, in patients from Japan and other Asian countries [8]. In addition, it has been suggested that the incidence of hypophosphatemic osteomalacia due to Fanconi syndrome associated with low-dose ADV therapy may be higher than previously thought [9].

In patients with ADV-induced Fanconi syndrome and/or hypophosphatemic osteomalacia, withdrawal or dose reduction of ADV should be performed immediately [5], and oral phosphate, calcium, or vitamin D_3_ should be added as necessary. In our patient, the dose of ADV was immediately reduced from 10 mg daily to 10 mg every other day. We also initiated treatment with calcitriol (1.0 μg/day) because our patient had hypophosphatemia and slightly low serum calcium and vitamin D_3_ levels. Generalized renal tubular injury caused by ADV inhibits 1α-hydroxylase activity with subsequent reduction of the 1,25-dihyroxyvitamin D_3_ level, leading to a decrease in intestinal calcium and phosphate absorption that can contribute to development of osteomalacia [3]. However, generalized bone pain was not relieved in our patient, and several bone resorption markers remained very high, despite ADV dose reduction and vitamin D_3_ supplementation. The serum level of bone-specific ALP also remained high.

Interestingly, our patient had persistent elevation of bone resorption despite receiving treatment for osteomalacia. Previous studies have shown that bone resorption is occasionally increased in patients with hypophosphatemic osteomalacia because osteoclasts are unable to resorb nonmineralized osteoid [10]. Accordingly, our patient may have a mixed form of osteoporosis and osteomalacia (i.e., osteoporomalacia) [11]. Therefore, we added denosumab (anti-RANKL monoclonal antibody) to vitamin D_3_ supplementation in order to suppress bone resorption and treat his generalized bone pain. Two months after starting denosumab therapy, the patient’s hip and knee pain showed improvement, together with a decrease in serum bone-specific ALP and bone resorption markers.

Denosumab is a human immunoglobulin G2 monoclonal antibody that inhibits bone resorption by targeting RANKL, which is involved in osteoclast differentiation [4]. Several studies have demonstrated that denosumab is effective for reducing the risk of fracture in women with postmenopausal osteoporosis [12]. Unlike bisphosphonates (another class of potent antiresorptive agents), denosumab does not cause more adverse events in patients with impaired kidney function, because renal insufficiency does not affect its pharmacokinetics or pharmacodynamics [13]. Thus, we chose denosumab for our patient because he had Fanconi syndrome with generalized proximal tubular dysfunction caused by ADV therapy.

In general, antiresorptive agents such as bisphosphonates and denosumab may not be appropriate for treating hypophosphatemic osteomalacia or other forms of osteomalacia, regardless of the degree of renal insufficiency and vitamin D level. Severe and prolonged hypocalcemia was reported after a single injection of denosumab (60 mg) in a patient with osteomalacia due to Fanconi syndrome [14], because calcium homeostasis is dependent on high bone turnover in osteomalacia. Monitoring of the serum calcium level also is mandatory to prevent severe hypocalcemia when denosumab is initiated in all forms of osteomalacia. Very recently, there was another case report of hypophosphatemia osteomalacia secondary to Fanconi syndrome in which bone pain was worsened by administration of denosumab [15]. Unlike in these reports, we observed that 2 months after initiation of denosumab, hip and knee pain of our patient was relieved along with a decrease in serum ALP and some bone resorption markers. We speculate that denosumab had worked well in our patient because of an adequate administration of vitamin D_3_ prior to denosumab. We speculate that denosumab may be an option for patients who have hypophosphatemic osteomalacia due to ADV-induced Fanconi syndrome with concurrent enhancement of bone resorption and/or osteoporosis. However, clinicians should keep in mind that if denosumab is administered to patients with hypophosphatemic osteomalacia accompanied by persistent excessive bone resorption despite treatment for osteomalacia, adequate vitamin D and/or phosphate supplementation should be done before administration of denosumab.

## Abbreviations

ADV: Adefovir dipivoxil
AG: Anion gap
Alb: Albumin
ALP: Alkaline phosphatase
ALT: alanine aminotransferase
AST: aspartate aminotransferase
BCE: Bone collagen equivalents
BE: Base excess
BJ protein: Bence-Jones protein
BUN: Blood urea nitrogen
CH50: Total hemolytic complement
ChE: Cholinesterase
Cre: Creatinine
CRP: C-reactive protein
eGFR: Estimated glomerular filtration rate
FEUA: Fractional excretion of uric acid
FGF23: Fibroblast growth factor 23
gGTP: γ-Glutamyl transpeptidase
Hb: Hemoglobin
HbA1c: Hemoglobin A1c
HBe-Ab: Hepatitis B e antigen antibody
Hbs-Ag: Hepatitis B surface antigen
HCO_3_^−^: Bicarbonate
Hct: Hematocrit
Ig: Immunoglobulin
LAP: Leukocyte alkaline phosphatase
LDH: Lactate dehydrogenase
NTx: Cross-linked N-telopeptide of type I collagen
PCO_2_: Partial pressure of carbon dioxide
Plt: Platelets
PO_2_: Partial pressure of oxygen
PTH: Parathyroid hormone
PTHrP: Parathyroid hormone-related protein
RANKL: Receptor activator of nuclear factor-κB ligand
RBC: Red blood cells
T-Bil: Total bilirubin
TP: Total protein
TRACP-5b: Tartrate-resistant acid phosphatase 5b
%TRP: Percentage tubular reabsorption of phosphate
UA: Urinalysis
WBC: White blood cells
